# Hospitals of To-Day

**Published:** 1911-05-13

**Authors:** 


### HOSPITALS OF TO-DAY.

### V.—THE CANCER HOSPITAL AND THE PUBLIC.

The coming opening by the Duke of Connaught of the newly-built Research Institute in the grounds of the Cancer Hospital will mark the latest development in the activity of this institution. It is a well-known fact that the constant support which the Cancer Free Hospital has received in the past has allowed it not merely to acquire a site, likely to be sufficient for its needs for a considerable time, but to build on that site the extensions of which the new nurses' home, opened last year, and the present institute are the latest examples. In this respect the history of the hospital makes more encouraging reading than that of most institutions. The disease which it studies is one for the treatment of which the public subscribes eagerly, and a cancer hospital shares with a hospital for children and a consumptive sanatorium the highest popularity among charities. It is fortunate that this is so, for, as everyone knows, the methods of treatment now advocated for cancer are the most expensive branch of therapeutics, which is one reason why the cost per bed is usually higher in cancer than in general or other special hospitals. Another reason is that where, as at the Cancer Hospital, the number of beds to the ward is small compared with the old forty or even the modern twenty, the cost of administration is also increased, or, if it is preferred to say so, fewer patients are warmed and waited upon for an equal sum of money.

Bearing these simple facts in mind, we see how it is that the Cancer Hospital has probably received as large a share of legacies as any hospital, why the assistance of King Edward's Fund has been dispensed with, and why, at the same time, many of these legacies cannot be placed to the capital account, but are diverted to current income. At the present moment, for instance, an extension of the radium treatment is proposed, and the purchase of £3,000 worth of radium is in contemplation. As The Hospital pointed out on March 11, the Electrical and Radio-Therapeutic Department is claiming a large share of expenditure, and the medical officer in charge, encouraged no doubt by what has been done for research in the new institute, is eagerly pressing the claims of his department on the secretary, Mr. Howell. A sub-committee has been sitting to decide the best methods of extending the electrical department, which, with whatever fittings it may be supplied, will almost certainly be enlarged.

One well-known feature of this hospital is the provision it has made for incurable cases. As will be expected, the demand on the relatively few beds at the disposal of incurable cases is large and urgent, and a plan shortly to be put in force will, it is hoped, extend this branch of accommodation. It will thus be seen that if this is a rich hospital, it is rich in projects for extending its utility.

The ordinary man has no idea of the extent to which gardens form part of present-day hospital sites, even in London. He knows nothing of the open ground at the back of the Great Ormond Street Hospital for Children, of the green spaces behind the Prince of Wales' Hospital, Tottenham, of the quadrangle and tennis court at the London Hospital, for instance. To this haphazard list must be added the charming inner court at the Cancer Hospital, which neither the addition of the nurses' home nor of the new institute has been able to absorb. They are serious encroachments, and have followed each other rapidly, but, let us hope, the garden after this will suffer no more. Such square gardens, however, have more than æsthetic value to the hospitals which they beautify; for in most cases they imply ownership of the land on the three surrounding sides, and such ownership is a characteristic feature at the Cancer Hospital. The row of houses on the left as you stand facing the nurses' home has now capitulated, for there are only two houses remaining in it whose lessees are not the hospital's tenants. Sufficient land to allow reasonable extension is found often where a square garden is the centre of the site.

At the end of this month the Cancer Hospital concludes the first year of its history as a corporate body, for its Royal Charter was granted in May last year. Its position, of course, has been still further strengthened by incorporation. It is no longer an association the members of which can only sue or be sued individually. It may sue in a court of law in its corporate person. It has the antique privilege of employing its own seal. There can be no doubt, in short, that the hospital will now be able to do more for cancer research, and has been placed in a position by which the yearly growing sums now spent on that research may be more easily administered and available.

This general outline of the present position in which the hospital finds itself may be borne out by a glance at last year's accounts, which shortly will be in the hands of subscribers. The yearly expenditure is something over £17,000, and, as will be expected, the principal object of the extraordinary expenditure is the maintenance of the Cancer Research Institute, which costs £2,700. On the supposition that no legacies were received last year, or that all legacies were placed to the capital account, the hospital was £2,075 short on its receipts. The remainder of its income is principally divided between the sums which it derives from general donations (£6,331), and the sums received from its invested property (£6,367). The legacies received during the year amounted to £12,000.

It is dangerous for any hospital to-day to trust too much to legacies. Few have to do so; and such special hospitals as the Cancer cannot always expect that the impulse to spend largely on research will be answered by an equally strong impulse on the part of the public to subscribe to it. There are fashions in such things, and different branches of scientific research succeed each other in popular estimation as to their desirability. The present position of the hospital is a strong one, in spite of its deficit, and in spite of the new sums for maintenance which the new institute will demand next year.

